# Synergistic anti-HIV-1 activity of griffithsin with tenofovir, maraviroc and enfuvirtide

**DOI:** 10.1186/1742-4690-8-S2-O36

**Published:** 2011-10-03

**Authors:** Geoffrey Férir, Kenneth E Palmer, Dominique Schols

**Affiliations:** 1 Rega Institute for Medical Research, Katholieke Universiteit Leuven, Leuven, Belgium; 2 Department of Pharmacology and Toxicology and Owensboro Cancer Research Program, James Graham Brown Cancer Center, University of Louisville School Of Medicine, Louisville, USA

## Background

More than 60% of the total HIV-1 infections worldwide are dominated by clade B and C. To stop the epidemic, effective prevention methods (e.g. microbicidal gel) are of extreme importance. Carbohydrate binding agents (CBAs) are very good microbicide candidates. We previously showed that various CBAs act synergistic with tenofovir against HIV-1 [1].

Griffithsin (GRFT), isolated from the red alga *Griffithsia* sp., shows very potent and broad-spectrum anti-HIV activity and recombinant forms can easily be produced [2,3].

## Materials and methods

HIV-1 replication was measured in MT-4 cells and peripheral blood mononuclear cells (PBMCs) by MTS method and p24 Ag ELISA respectively. Synergism was calculated using CalcuSyn software (Biosoft, Cambridge, UK) based on the median effect principle [4]. Combination indices (CI) < 0.9 are synergistic, 0.9 < CI < 1.1 are additive and CI > 1.1 are antagonistic.

## Results

We evaluated combinations of GRFT against HIV-1 clade B and clade C isolates with various glycosylation patterns on the viral envelope in PBMCs and MT-4 cells. In all combinations tested against clade B viruses BaL (R5) and NL4.3 (X4), GRFT showed synergistic activity with tenofovir, maraviroc, AMD3100 and enfuvirtide, based on the median effect principle with combination indices (CI) varying between 0.34 and 0.64 at the calculated EC_95_-level. Against the clade C viruses, ZAM18, DJ259 and ETH2220 (all R5), Cl-values varied between 0.70 and 0.79. The CI correlated with increased antiviral activity of each individual compound.

## Conclusions

The evaluated two drug combinations increase their antiviral potency and support further clinical investigation and evaluation in preexposure prophylaxis in the context of HIV-1 clade B and clade C infections. Difference in glycosylation motifs in gp120 have little, if any, effects on the antiviral activity of GRFT.
